# *Borrelia burgdorferi* (*sensu lato*) in ectoparasites and reptiles in southern Italy

**DOI:** 10.1186/s13071-019-3286-1

**Published:** 2019-01-15

**Authors:** Jairo Alfonso Mendoza-Roldan, Vito Colella, Riccardo Paolo Lia, Viet Linh Nguyen, Darci Moraes Barros-Battesti, Roberta Iatta, Filipe Dantas-Torres, Domenico Otranto

**Affiliations:** 1 Deparment of Veterinary Medicine, University of Bari, 70010 Valenzano (BA), Italy; 2 Faculty of Veterinary Medicine, University of São Paulo, São Paulo, 05508-270 Brazil; 3 Butantan Institute, São Paulo, 05503-900 Brazil; 4 Department of Veterinary Pathology, Universidade Estadual Paulista, Jaboticabal, 14884-900 Brazil; 5 Aggeu Magalhães Institute, Oswaldo Cruz Foundation, Recife, Pernambuco 50670-420 Brazil

**Keywords:** Reptiles, Ectoparasites, *Borrelia lusitaniae*, *Borrelia garinii*, *Ixodes ricinus*, *Podarcis siculus*

## Abstract

**Background:**

*Borrelia burgdorferi* (*sensu lato*) is a complex containing pathogenic bacteria of which some species, such as *Borrelia lusitaniae*, use birds, small mammals and reptiles as reservoirs. In Italy, the bacteria have been detected in reptilian and avian reservoirs in the northern and central regions.

**Results:**

Here, 211 reptiles from three orders [Squamata (Sauria with seven species in five families and Ophidia with 11 species in three families), Crocodylia (one family and two species), and Testudines (two families and two species)] were examined for ectoparasites and molecular detection of *B. burgdorferi* (*s.l.*) in three different sites of southern Italy, an area for which no information was previously available on the occurrence of borreliosis in animals and humans. *Borrelia lusitaniae* was molecularly detected in larvae and nymphs (11.6%) of *Ixodes ricinus* infesting lizards (i.e. *Podarcis muralis*, *Podarcis siculus* and *Lacerta bilineata*) and in 12.3% blood samples of *P. siculus*. Finally, *B. lusitaniae* and *Borrelia garinii* were detected in 5.1% (32/630) of questing *I. ricinus*.

**Conclusions:**

These results show the circulation of *B. lusitaniae* in southern Italy and suggest that *P. siculus* could play a role as a reservoir, representing a potential medical threat to humans living in or visiting these localities.

## Background

The genus *Borrelia* comprises spirochete bacteria distinguished in the relapsing fever, the reptilian *Borrelia*, monotreme associated *Borrelia*, and the Lyme borreliosis groups [1, 2]. The latter includes around 20 species within the *Borrelia burgdorferi* (*sensu lato*) complex, nine of which with recognized pathogenic outcomes to animals and/or humans (i.e. *Borrelia afzelii*, *Borrelia bavariensis*, *Borrelia bissettii*, *B. burgdorferi* (*s.s.*), *Borrelia garinii*, *Borrelia kurtenbachii*, *Borrelia lusitaniae*, *Borrelia spielmanii* and *Borrelia valaisiana*) in Palaearctic and Nearctic regions [3, 4].

In Europe, more than 232,000 new cases of Lyme borreliosis are reported each year in human patients [5–8]. However, there are a significant amount of unreported cases [3], mainly due to the pleomorphic clinical presentation of the infection, which ranges from unspecific flu-like symptoms to typical Lyme disease clinical sings (e.g. *erythema migrans* and other dermatological signs, carditis, chronic arthritis, meningitis and neurological abnormalities) [9]. These bacteria are mainly transmitted by hematophagous arthropods, such as ticks, with nymphs of the castor bean tick, *Ixodes ricinus*, and other species, such as *Ixodes persulcatus*, vectors of the pathogens in Europe [3, 10, 11]. The harvest mite *Neotrombicula autumnalis* is widely distributed in Europe and has been also investigated as potential vector [12]. However, current knowledge indicates that *Ixodes* spp. (e.g. *I. ricinus*, *Ixodes scapularis*, *I. persulcatus* and *Ixodes pacificus*) [13] ticks are the main vectors of these pathogens since their immature stages feed on the reservoirs for these spirochetes (i.e. small mammals, passerine birds and reptiles, especially lacertid lizards) [14]. For example, distinct species of lacertid lizards have been pointed out as reservoirs of *B. lusitaniae* throughout Europe [15]. This pathogen has been isolated from human patients and associated to chronic skin lesions and vasculitis-like syndrome, first in Portugal and thereafter in other Mediterranean regions [16, 17]. Additionally, *B. lusitaniae* has been detected in ticks and lacertid lizards such as *Psammodromus algirus* in Tunisia, *Lacerta agilis* in Slovakia, and *Podarcis muralis* and *L. agilis* in Germany [18–21]. In Italy, *B. lusitaniae* and other species of the *B. burgdorferi* (*s.l.*) have been detected in ticks and in birds, small native and invasive rodents, and lizards in Tuscany (central region) and Piedmont and Veneto (northern region) [22–24]. The common and highly synanthropic wall lizard (*P. muralis*) has been indicated as an important natural reservoir for its wide distribution [25]. Moreover, while no information was previously available on the occurrence of borreliosis in southern Italy, sporadic cases of Lyme borreliosis have been unofficially reported in this geographical area. In this study, we investigated the presence of *B. burgdorferi* (*s.l.*) in mites and ticks, as well as in blood and tissue samples from reptiles in southern Italy, to assess the circulation of these bacteria in reptiles and their ectoparasites and, therefore, the potential risk for human infections.

## Methods

### Study areas

The study was conducted in two areas of the Apulia region (site 1 and 2), and one of the Basilicata region (site 3), southern Italy. In Apulia, collections were performed in the surrounding areas of the Department of Veterinary Medicine, University of Bari “Aldo Moro” (41°1'31.584"N, 16°54'3.6288"E), in the province of Bari (site 1). This site is inhabited by some species of synanthropic reptiles (*P. muralis*, *P. siculus*, *Tarentola mauritanica*, *Hierophis carbonarius*, *Natrix natrix* and *Elaphe quatuorlineata*) [26, 27]. Also, exotic captive-bred reptiles (Ophidia and Testudines) owned by faculty members that lived in Valenzano municipality were examined for ectoparasites. Site 2 was a zoological park in Fasano (province of Brindisi; 40°49'56.8236"N, 17°20'26.7612"E) where more than 100 reptile species, both endemic and exotic, are exposed to ectoparasites. Finally, in the Basilicata region, collections were performed in the Gallipoli Cognato Forest (site 3; 40°32'17"N, 16°07'20.17"E), which belongs to the Parco Regionale di Gallipoli Cognato Piccole Dolomiti Lucane. This park is located in a region with a high diversity of herpetofauna, including eight common species of snakes (*H. carbonarius*, *N. natrix*, *Natrix tessellata*, *Coronella austriaca*, *E. quatuorlineata*, *Zamenis lineatus*, *Zamenis situla* and *Vipera aspis*), one species of tortoise (*Testudo hermanni*) and six species of lizards (*L. bilineata*, *P. mularis*, *P. siculus*, *T. mauritanica*, *Hemidactylus turcicus* and *Mediodactylus kotschyi*) [26]. The collection sites were chosen based on previous studies on the occurrence and seasonality of questing ticks from the environment [28], and for the occurrence of unofficial reports of human cases of borreliosis (site 3).

### Reptile capturing and processing and ectoparasites collection

Lizards and snakes were captured by hand and herpetological hooks at different sites from March to August 2018 (i.e. spring and summer). Species of reptiles were identified using reference keys [29], and the ticks and mites were removed by scarification methods and stored in 70% ethanol. In lizards, a small amount of blood was obtained *via* tail fracture, or by cardiocentesis, when animals were adults and non-gravid females. Blood samples were stored at -20 °C and tail tissue in 70% ethanol. In larger reptiles, such as snakes and larger Sauria, blood samples were obtained from the ventral coccygeal vein and stored in EDTA tubes, later they were transferred to 1.5 ml tubes and stored at -20 °C.

A total of 630 *I. ricinus* ticks collected under the frame of a previous study [28] were included in this investigation. In brief, ticks were collected from site 3 by dragging and flagging in the same areas where the reptiles were captured. *Borrelia burgdorferi* (*s.l.*) was molecularly detected by using protocols below. Ticks and mites were identified to the species level using dichotomic identification keys [30–34].

### Detection of *B. burgdorferi* (*s.l.*) DNA

DNA was extracted from mites and ticks using a modified guanidine isothiocyanate protocol, which allowed the preservation of a voucher [35, 36], and from tail tissue (25 mg) from lizards, snakes and other reptiles, by using a Qiagen DNeasy tissue kit (Qiagen, Hilden, Germany). DNA was suspended in Buffer AE (elution buffer) (50 μl for mites, ticks and tissues) and used in PCR (2 μl per reaction). DNA was extracted from reptile blood (~20 μl) by using a Qiagen DNeasy blood mini kit (Qiagen).

The presence of *B. burgdorferi* (*s.l.*) flagellin gene in mites, ticks and tissues was investigated by PCR, as described previously [37]. DNA samples from ticks positive to *B. afzelii* detected in a previous study [38] were used as positive controls. For PCR-negative ticks, the efficiency of extraction protocol was verified by amplifying the *16S* rDNA fragment of ticks [39].

Amplified DNA was subjected to electrophoresis in a 2% agarose gel stained with GelRed (VWR International PBI, Milano, Italy) and viewed on a GelLogic 100 gel documentation system (Kodak, New York, USA). Amplicons were purified using 10 μl of PCR product mixed with 0.5 μl of *Escherichia coli* exonuclease I (Exo I; MBI, Fermentas, Lithuania), 1 μl of shrimp alkaline phosphatase (SAP) and 0.5 μl of SAP reaction buffer (MBI, Fermentas, Lithuania) to remove primers and unincorporated dNTPs. This mix was incubated at 37 °C for 20 min, following enzymes inactivation at 85 °C for 15 min. PCR purified products were sequenced using the Taq DyeDoxyTerminator Cycle Sequencing Kit (v.2, Applied Biosystems) in an automated sequencer (ABI-PRISM 377). Sequences were analyzed by Geneious version 11.1.4 software and submitted to BLAST to identify similarities to known sequences [40].

To confirm the morphological identification of ticks, amplified sequences were aligned using ClustalW [41] and with the corresponding mitochondrial *16S* rDNA sequences of *I. ricinus* (GU074612, KR870982, GU074594, KX384806, GU074592, GU074595, KM211788, GU074596, KM211785 and KM211787) and *Ixodes inopinatus* (KM211790 and KM211789). In addition, sequences of other *Ixodes* species available in the GenBank database were included and used as outgroups. Phylogenetic analyses were carried out using the maximum likelihood (ML) method with the program MEGA 5 [42]. The ML tree was generated with the Hasegawa-Kishino-Yano model by using a discrete gamma-distribution (+G). The best-fitting substitution models were determined with the Bayesian Information Criterion using the ML model test. Support was tested with 2000 bootstrap pseudoreplicates.

### Statistical analysis

To assess the parasitic load of ticks, descriptive statistics was calculated using Quantitative Parasitology software, version 3.0 [43]. Prevalence, mean abundance (number of ticks per total number of hosts) and mean intensity (number of ticks per number of infested hosts) of infestation were determined.

## Results

**Fig. 1:**
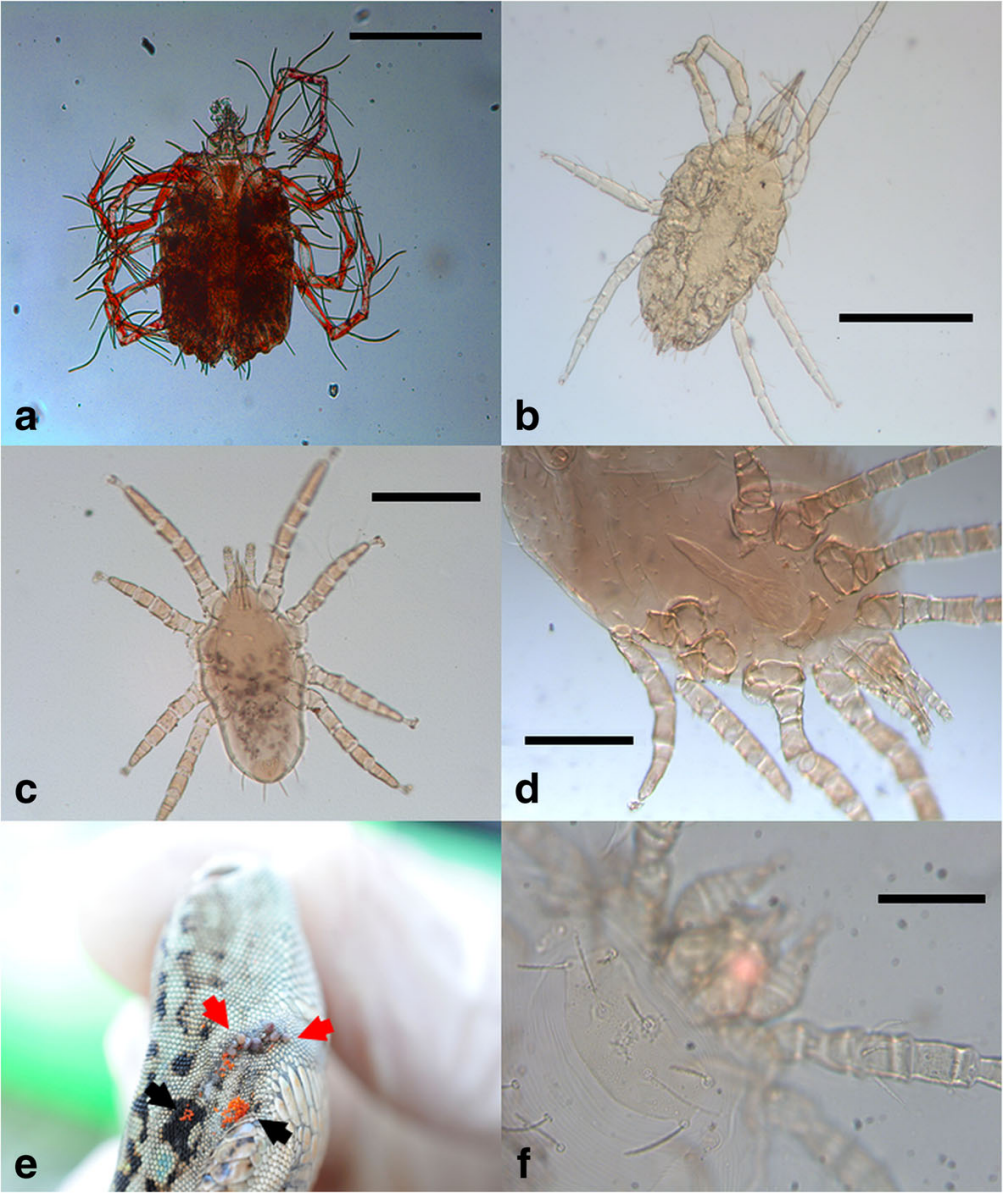
Mite species found on reptiles from southern Italy. **a** *Pimeliaphilus insignis* on *Tarentola mauritanica*. **b** *Geckobiella stamii* on *Iguana iguana*. **c** Deutonymph of *Ophionyssus sauracum* on *Podarcis siculus*. **d** *Ophionyssus natricis* on *Eunectes murinus*. **e** *Podarcis siculus* co-infested with larvae of *Neotrombicula autumnalis* (black arrows) and immature stages of *Ixodes ricinus* (red arrows). **f** Larva of *Neotrombicula autumnalis*. *Scale-bars*: **a**, **b**, 500 μm; **c**, 200 μm; **f**, 50 μm

**Fig. 2:**
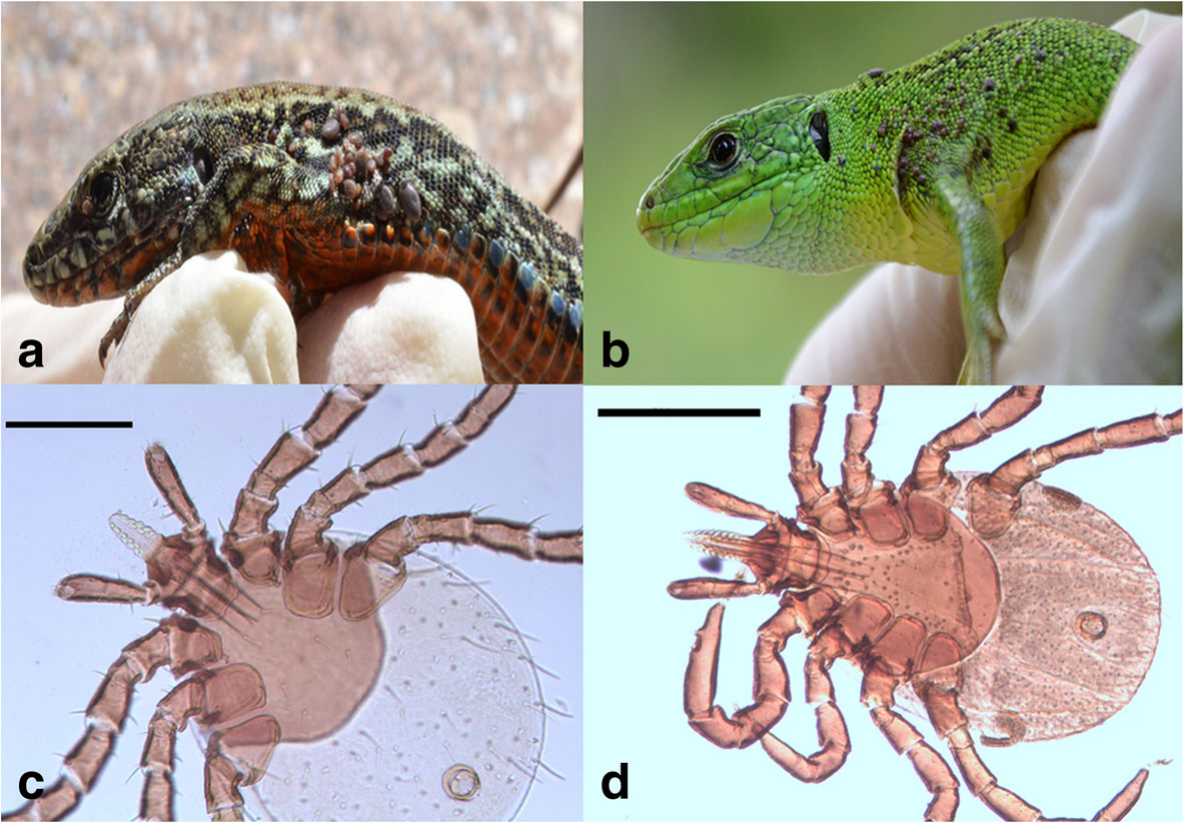
Immature stages of *Ixodes ricinus* ticks on lacertid lizards. **a** *Podarcis muralis* infested with immature stages of *I. ricinus*. **b** *Lacerta bilineata* infested with immature stages of *I. ricinus*. **c** Voucher material of larva of *I. ricinus*. **d** Voucher material of nymph of *I. ricinus*. *Scale-bars*: **c**, 200 μm; **d**, 500 μm

Two hundred and eleven reptiles from three orders [Squamata (Sauria with seven species in five families and Ophidia with 11 species in three families), Crocodylia (one family and two species), and Testudines (two families and two species)] (*n* = 56 in site 1, *n* = 23 in site 2, and *n* = 134 in site 3) were examined, of which 174 were infested by mites and ticks (82.5%; 95% CI: 76.6–87.3%). Specifically, 67.8% (95% CI: 54–79.7%) and 39.1% (95% CI: 19.7–61.4%) of reptiles from site 1 and 2, respectively, were infested solely by mites, whereas 94.8% (127/134; 95% CI: 89.5–97.8%) of animals in site 3 were infested by mites and ticks (Tables 1, 2). Two species of Pterygosomatidae (*Pimeliaphilus insignis* and *Geckobiella stamii*) (Fig. 1a, b), one species of Trombiculidae (*Neotrombicula autumnalis*) (Fig. 1e, f) and two species of Macronyssidae (*Ophionyssus sauracum* and *Ophionyssus natricis*) were identified (Fig. 1c, d) (Table 2). *Ixodes ricinus* was the only tick species detected (Fig. 2). Different host species were co-infested with *N. autumnalis* and *O. sauracum*, or with *I. ricinus* and *N. autumnalis* (Fig. 1e, Table 2). A total of 654 *I. ricinus* (449 larvae and 205 nymphs) (Fig. 2c, d) were collected from 120 reptiles from site 3 (90.9%; 95% CI: 84.6–95.2%) (*L. bilineata*, *P. muralis*, *P. siculus* and *E. quatuorlineata*) with a mean intensity of 5.5 (95% CI: 5.4–6.2%) and a mean abundance of 5 (95% CI: 4.45–5.6%).

**Table 1:** Species of reptiles examined in the study area and their infestation rates (no. infested/no. examined)

Family	Species	Site 1	Site 2	Site 3	Total no. infested
Lacertidae	*Podarcis muralis*	0/1	***–***	17/17	17
	*Podarcis siculus*	29/29	***–***	102/106	131
	*Lacerta bilineata*	***–***	***–***	5/5	5
Gekkonidae	*Tarentola mauritanica*	9/10	***–***	2/2	11
Iguanidae	*Iguana iguana*	***–***	2/2	***–***	2
Teiidae	*Salvator merianae*	***–***	0/1	***–***	0
Scnicidae	*Tiliqua* sp.	***–***	0/1	***–***	0
Alligatoridae	*Paleosuchus palpebrosus*	***–***	0/1	***–***	0
	*Caiman crocodilus*	***–***	0/1	***–***	0
Boidae	*Boa constrictor*	0/4	3/3	***–***	3
	*Eunectes murinus*	***–***	1/1	***–***	1
Pythonidae	*Python molurus*	***–***	0/1	***–***	0
	*Python regius*	0/9	0/2	***–***	0
	*Morelia spilota*	***–***	3/4	***–***	3
Colubridae	*Pituophis melanoleucus*	***–***	0/3	***–***	0
	*Panterophis obsoletus*	***–***	0/1	***–***	0
	*Panterophis guttatus*	0/1	0/1	***–***	0
	*Elaphe quatuorlineata*	-	***–***	1/2	1
	*Natrix natrix*	***–***	***–***	0/1	0
	*Hierophis carbonarius*	***–***	***–***	0/1	0
Chelydridae	*Chelydra serpentina*	***–***	0/1	***–***	0
Emydidae	*Trachemys scripta*	0/2	***–***	***–***	0
Total		38/56	9/23	127/134	174

**Table 2:** Species of Acari identified on different host species (all free living otherwise indicated) and collection sites

Family	Species	Host	Collection site^a^
Pterygosomatidae	*Pimeliaphilus insignis*	*T. mauritanica*	Site 1 and Site 3
	*Geckobiella stamii*	*I. iguana*	Site 2
Trombiculidae	*Neotrombicula autumnalis*	*P. muralis*, *P. siculus*, *L. bilineata*	Site 1 and Site 3
Macronyssidae	*Ophionyssus sauracum*	*P. muralis*, *P. siculus*, *L. bilineata*	Site 1 and Site 3
	*Ophionyssus natricis*	*M. spilota*, *E. murinus*, *B. constrictor*	Site 2
Ixodidae	*Ixodes ricinus*	*P. muralis*, *P. siculus*, *L. bilineata*, *E. quatuorlineata*	Site 3

^a^All host species from Fasano (site 2) were captive

Of the ectoparasite species collected from reptiles, only *I. ricinus* ticks scored positive for *B. burgdorferi* (*s.l.*), with an overall prevalence of 11.6% (95% CI: 7.9–16.4%). Specifically, 3.6% (95% CI: 1.1–8.1%) of larvae and 21.8% (95% CI: 14.5–30.7%) of nymphs tested positive for the spirochetes DNA. In site 3, a high percentage of reptiles carried infected *B. burgdorferi* (*s.l.*) ticks (Table 3) as larvae (i.e. 11.9% of *P. siculus*), and/or nymphs (i.e. 31.7% of *P. siculus*, 33.3% of *P. muralis* and 60% of *L. bilineata*). None of the tail tissue samples from 156 lizards and 12 geckos scored positive to *B. burgdorferi* (*s.l.*), whereas blood samples from 14 (12.3%; 95% CI: 6.8–19.7%) *P. siculus* lizards from site 3 were positive. In particular, 25.9% (95% CI: 14.9–39.6%) of blood samples collected from lizards and snakes infested with ticks in site 3 were positive to *B. burgdorferi* (*s.l.*). Overall, 18.5% (95% CI: 6.3–38%) of *B. burgdorferi* (*s.l.*)-infected ticks were collected from infected lizards. When both blood and tail from the same reptile were processed, *B. burgdorferi* (*s.l.*) was detected only in the former tissue (i.e. 14/84 in blood *vs* 0/84 in tails). DNA sequences obtained from immature engorged ticks collected from reptiles (28 sequences) and blood (14 sequences) were identified as *B. lusitaniae* (100% nucleotide identity with GenBank sequence KY213886).

**Table 3:** Molecular detection of *B. burgdorferi* (*s.l.*) in immature stages of *I. ricinus* and in the associated reptile host species

Host species	No. of infested individuals	No. of ticks tested	No. of positive hosts/ ticks	Hosts with *B. burgdorferi* (*s.l.*)-infected ticks (95% CI) (%)		Ticks infected with *B. burgdorferi* (*s.l.*) (95% CI) (%)	
				Larvae	Nymphs	Larvae	Nymphs
*E. quatuorlineata*	1	8	0/0	0	na	0	na
*P. siculus*	102	180	14/23	11.9 (3.9–25.6)	31.7 (20.2–44.9)	5.0 (1.64–11.2)	23.7 (14.9–34.5)
*P. muralis*	12	26	0/2	0	33.3 (4.3–77.7)	0	20.0 (2.5–55.6)
*L. bilineata*	5	36	0/3	0	60.0 (14.6–94.7)	0	15.0 (3.2–37.9)

*Abbreviation*: *na* not applicable

**Fig. 3:**
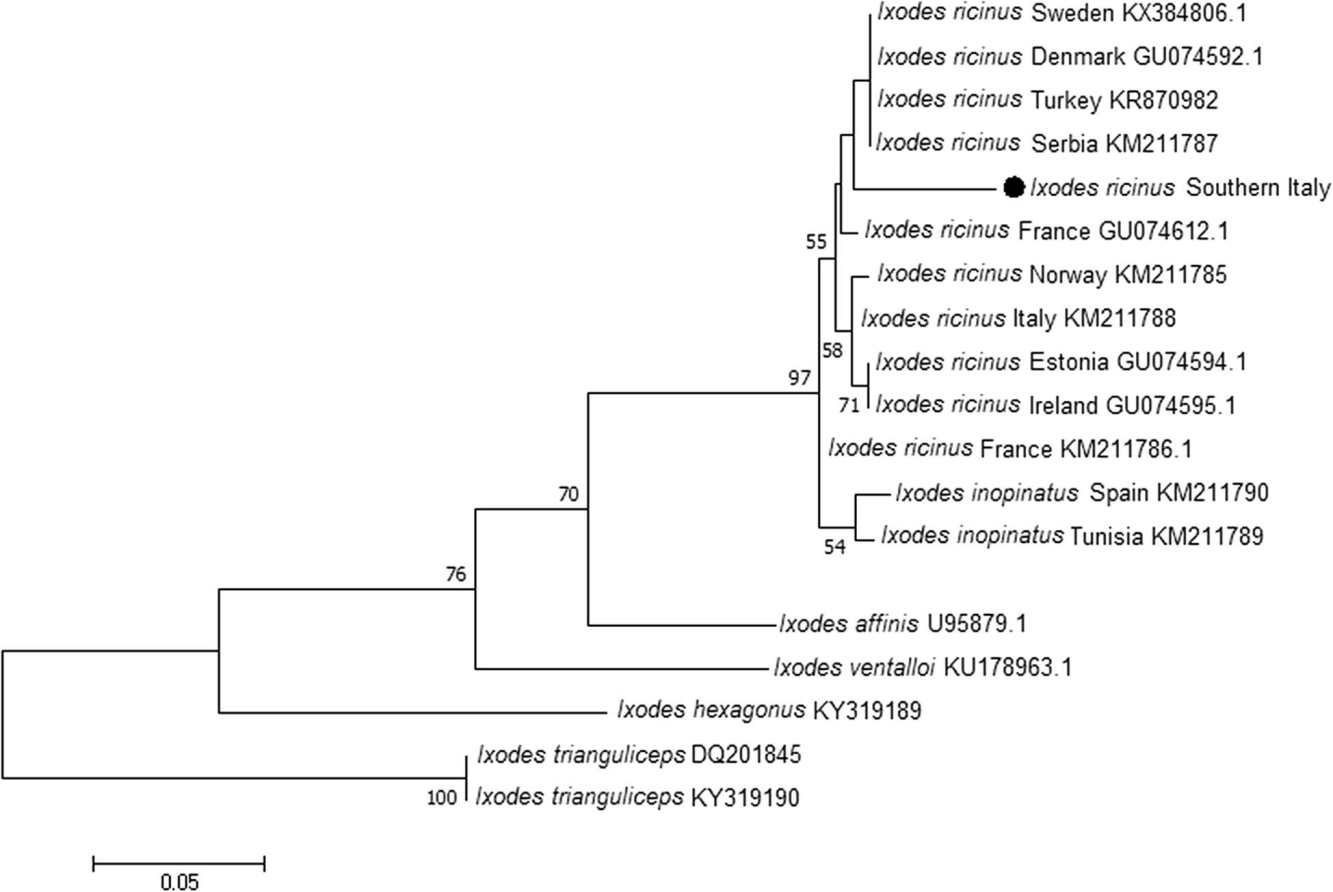
Phylogenetic tree based on maximum likelihood analysis of the mitochondrial *16S* rDNA sequences obtained from specimens of *I. ricinus* (consensus sequence of this study is highlighted with a circle) collected in southern Italy and sequences from specimens of *I. inopinatus* KM211789 (*I. inopinatus*, Tunisia), KM211790 (*I. inopinatus*, Spain). Other sequences included of *I. ricinus* from Europe and northern Africa are available on GenBank. *Ixodes affinis* (U95879)*, Ixodes ventalloi* (KU178963)*, Ixodes hexagonus* (KY319189), *and Ixodes trianguliceps* (DQ201845 and KY319190) were used as outgroups

A total of 630 questing adults (144 females and 181 males) and nymphs (305) of *I. ricinus* were tested for *B. burgdorferi* (*s.l.*) and 5.1% (32/630; 95% CI: 3.4–7.1%) yielded positive results. Two different species, namely *B. lusitaniae* (31/32) and *B. garinii* (1/32), were identified with 100% and 99.9% nucleotide identity with GenBank sequence KX646194 and KR782222, respectively. The prevalence of *B. burgdorferi* (*s.l.*) was higher in nymphs 5.9% (18/305) than in adults 4.3% (14/325). Sequences obtained of the flagellin gene of *Borrelia* species were deposited in GenBank under the accession numbers MH751501, MH751502 and MH807255. The consensus sequences of mitochondrial *16S* gene of *I. ricinus* from this study (GenBank: MH751500) grouped with other sequences of the same species, distinct from those related to *I. inopinatus* with high bootstrap support (97%) (Fig. 3).

## Discussion

In this study, we identified Lyme borreliosis group species in *I. ricinus* ticks from the environment, and from blood samples and ticks from reptiles captured in three different locations in southern Italy. Free-living or captive reptiles were parasitized by a range of mites (five species belonging to three different families) and/or ticks. The latter can infest a wide number of vertebrate hosts including humans, and act as vectors of pathogens of medical and veterinary significance. For instance, *B. lusitaniae* was found in larvae and nymphs of *I. ricinus* (11.6%) collected from three species of lacertid lizards, in blood of *P. siculus* lizards (12.3%) and in questing *I. ricinus* adults and nymphs (5.1%)*.* These results, together with the high infestation rates of *I. ricinus* on lizards, suggest that these reptiles may contribute in maintaining *B. lusitaniae* endemicity in southern Italy. Although the role of lizards as natural reservoirs of *B. burgdorferi* (*s.l.*) has been questioned [44, 45], previous reports from endemic regions, such as Czech Republic, Slovakia, Poland and Romania, suggest the participation of these reptiles in the ecology of *B. lusitaniae* [22–25, 46]. The prevalence of *B. lusitaniae* in ticks from lizards (11.6%) is higher than that reported in Poland (6%), lower than that in Slovakia (21%), and similar to that in Romania (13%) [20]. Noteworthy, the prevalence of *B. lusitaniae* in ticks in southern Italy, where no official cases of borreliosis are reported, is higher than that recorded in Piedmont (4%), northern Italy, an area where human Lyme borreliosis is endemic [23]. The finding of *B. lusitaniae* in *P. siculus* suggests that this species may act as a reservoir for this spirochete, as demonstrated for *P. muralis* [22]. However, since tail tissue (poor in muscle, connective tissue and highly keratinized) is not the optimal sample for the detection of *Borrelia* spp. infection [20], the results of this study may have been underestimated. Accordingly, the positivity rate could have been also affected by the intermittent nature of *Borrelia* bacteremia. The finding of positive tick nymphs in negative *L. bilineata* and *P. muralis* lizards may suggest that they fed previously on other positive hosts as larvae. The higher number of positive nymphs compared to larvae indicates that the latter have been infected feeding on other reservoirs (e.g. *P. siculus* lizards, small mammals or birds) and as nymphs on other lacertid lizards. In addition, co-feeding ticks (i.e. feeding at the same site) can favor the spreading of the bacteria, as supported by molecular detection of the pathogen when testing skin biopsies at tick attachment sites [47]. Hence, lizards may play a more significant role than mice in the maintenance and transmission of *B. lusitaniae* [22, 23], whereas rodents are the main reservoirs of *B. afzelii* in Germany, England and Slovakia [18, 48, 49]. This variation of reservoirs and prevalence of infection among ticks and hosts can be explained by the relative abundance of vertebrate hosts in selected geographical area.

Although other reptiles have been found to harbor pathogenic and non-pathogenic borrelial species [1, 50, 51], we did not find any positive non-lacertid reptile. The overall prevalence of borrelial infections (5.1%) in questing ticks was lower compared to that recorded in other areas (19% in Slovakia, 20.5% in Czech Republic, and 16.7–39% in Tuscany, central Italy) [20, 52, 53]. In Italy, borrelial species have been detected in questing ticks with a lower prevalence in the northern region (1.4–3.8%) than in the central region (17.6%) and in urban or peri-urban areas [25, 54, 55]. Generally, the presence of *Borrelia* spp. in ticks increases through their life stages due to the wide number of hosts they can feed on [56]. Different from other studies, the positivity in questing ticks was higher in nymphs (5.9%), than in adults (4.3%). Although this difference was not significantly higher, this pattern could change due to seasonal variations and hosts availability [57]. In addition, *B. garinii* was herein detected in a questing tick and, before, in ticks from passerine birds [58]. This species is associated with neuroborreliosis in humans, and birds are their main reservoirs [59].

The flagellin gene sequence of *B. lusitaniae* from this study was 99% identical to that of *B. lusitaniae* (KY213886) detected from *I. ricinus* infesting a dog in central Italy [52]. The distribution of *B. lusitaniae* can be associated to the presence of highly synanthropic lizards inhabiting the Mediterranean area, which features a variety of environments (e.g. dry and warm or humid and cold). Indeed, due to their high biological plasticity, lacertid lizards occupy diverse habitats facilitating the dispersal of the tick infestation and, therefore, of borrelial infection [60]. Although *I. inopinatus* has been reported from dry areas of the Mediterranean region parasitizing mainly lizards [34], we identified only *I. ricinus* on this host species, in accordance with previous observations [61].

While most of the species of mites identified are commonly found on reptiles, some are exotic to Italy, such as *G. stamii*, which was reported for the first time in Italy in captive-owned *Iguana iguana* in 2016 [62]. Also, *O. natricis*, an exotic parasite widely distributed in captive reptiles, was herein found infesting snakes (i.e. *Morelia spilota*, *Boa constrictor* and *Eunectes murinus*), highlighting the risk associated with the introduction of exotic reptiles. Indeed, this mite can produce dermatitis, anemia and disecdysis, which were seen on the infested animals in site 2 (data not shown). In addition, *O. natricis* can infest humans and act as vector of reptile pathogens [63, 64]. Furthermore, the most common species of mite found on lizards in sites 2 and 3 was *N. autumnalis* larvae. Due to the transovarial transmission of *Anaplasma phagocytophilum* [65], and possibly of *B. burgdorferi* (*s.l.*) [12], *N. autumnalis* has been suggested to play a role as vector of these pathogens. Lizards highly infested by *N. autumnalis* did not show signs of detriment associated with the infestation, whereas in other animals and humans *N. autumnalis* can produce dermal lesions called *erythema autumnale* dermatitis, and conjunctivitis [66, 67]. Trombiculosis infestations are common in urban and peri-urban areas and synanthropic lizards can be propagators of this mite species, which can later parasitize mammalian hosts [68]. In this study, all *N. autumnalis* were negative for *B. burgdorferi* (*s.l.*), therefore suggesting that this species does not act as suitable vector in southern Italy. Accordingly, the rate of infection, the transovarian transmission and the transmission of the infection of *B. burgdorferi* (*s.l.*) by *N. autumnalis* was very low, thus indicating that this mite species has a minor role in the ecology of borreliosis [12]. Nonetheless, further investigations are needed to better understand the role of trombiculid mites in pathogen transmission among animals and humans.

## Conclusions

Results of this study revealed that *B. lusitaniae* circulates in southern Italy and indicated *P. siculus* as a potential natural reservoir for this species. Additionally, *B. garinii* was detected in a questing tick and, considering its pathogenicity, deserves further investigations. These results should increase medical awareness on the occurrence of *Borrelia* spp. in southern Italy to refine diagnosis and treatment of human borreliosis, thus facilitating official reports of clinical cases. Additional studies should elucidate the role of synanthropic lizard and other reptiles in the transmission of different *B. burgdorferi* (*s.l.*) species to humans.

## Abbreviations

CI: confidence interval
na: not applicable
*s.l.*: *sensu lato*
